# Surface-Enhanced Raman Scattering-Based Immunoassay Technologies for Detection of Disease Biomarkers

**DOI:** 10.3390/bios7010007

**Published:** 2017-01-12

**Authors:** Joseph Smolsky, Sukhwinder Kaur, Chihiro Hayashi, Surinder K. Batra, Alexey V. Krasnoslobodtsev

**Affiliations:** 1 Department of Physics University of Nebraska at Omaha 6001 Dodge Street, Omaha, NE 68182, USA; jsmolsky@unomaha.edu; 2 Department of Biochemistry and Molecular Biology, University of Nebraska Medical Center, Omaha, NE 68198, USA; skaur@unmc.edu (S.K.); chihiro.hayashi@unmc.edu (C.H.); sbatra@unmc.edu (S.K.B.)

**Keywords:** Surface-Enhanced Raman Scattering, biomarkers, nanodiagnostics, immunoassay

## Abstract

Detection of biomarkers is of vital importance in disease detection, management, and monitoring of therapeutic efficacy. Extensive efforts have been devoted to the development of novel diagnostic methods that detect and quantify biomarkers with higher sensitivity and reliability, contributing to better disease diagnosis and prognosis. When it comes to such devastating diseases as cancer, these novel powerful methods allow for disease staging as well as detection of cancer at very early stages. Over the past decade, there have been some advances in the development of platforms for biomarker detection of diseases. The main focus has recently shifted to the development of simple and reliable diagnostic tests that are inexpensive, accurate, and can follow a patient’s disease progression and therapy response. The individualized approach in biomarker detection has been also emphasized with detection of multiple biomarkers in body fluids such as blood and urine. This review article covers the developments in Surface-Enhanced Raman Scattering (SERS) and related technologies with the primary focus on immunoassays. Limitations and advantages of the SERS-based immunoassay platform are discussed. The article thoroughly describes all components of the SERS immunoassay and highlights the superior capabilities of SERS readout strategy such as high sensitivity and simultaneous detection of a multitude of biomarkers. Finally, it introduces recently developed strategies for in vivo biomarker detection using SERS.

## 1. Introduction

The use of Surface-Enhanced Raman Scattering (SERS) in sensing applications has recently received a lot of attention. Strong enhancements of Raman signals for molecules adsorbed on nanostructures made of noble metals such as silver and gold dramatically increase Raman scattering cross sections. SERS effect overcomes the inherent weakness of Raman spectroscopy, which is the low intensity of Raman signals. This effect combined with the characteristic molecular fingerprint of the Raman spectrum makes SERS a very promising candidate for various applications. In particular, SERS has been utilized in analytical studies, clinical diagnoses, environmental monitoring, and biomolecule detection.

Additionally, recent developments in nanoscience and nanotechnology allowing for more controllable manufacturing of nanostructures have contributed to the popularity of SERS in various strategies aimed at detection of molecular biomarkers. Early diagnosis and management of a disease heavily rely on biomarker detection in body fluids such as blood and urine. Body fluids are a complex mixture of biomolecules and the biomarkers are typically present at low concentrations, which makes biomarker detection very challenging. Since SERS can detect molecules of interest even at the single-molecule level, particular efforts have been dedicated to the development of biosensors operating via SERS. Specific attention has been given to SERS immunoassays based on antigen–antibody binding. While SERS provides high sensitivity, antibody–antigen interactions contribute to high specificity. The combination of the two ensures that SERS-based immunoassay detection platforms are suitable for low-level biomarker detection in early disease diagnosis. 

## 2. Sandwich Immunoassay Format

The ability to reliably detect low levels of specific biomarkers in body fluids provides an effective test for early diagnosis of a condition, predicting relapse and prognosis and assessing response to therapy. Disease detection at early stages drastically increases treatment success. There are several important key factors to consider in biomarker detection. One is that effective biomarker detection should involve body-fluid-based assays such as blood or urine. For example, a serum test is the most direct way to analyze the content of the patient biomarker pool and thus is the most used test for the detection of biomarkers in clinical settings. A major drawback of using serum samples is that they are complex fluids containing many various components besides targeted biomarkers. Therefore, the specificity of the detecting methodology should be very high. In addition to specificity, another desirable property of the effective diagnostic methodology is high sensitivity. Thus, sensors detecting biomarkers with high reproducibility at very low concentrations are required. One such promising sensing modality is immunoassay in a sandwich format. Detection based on the immunoassay format allows for sensitive and specific recognition of biomarkers.

**Figure 1:**
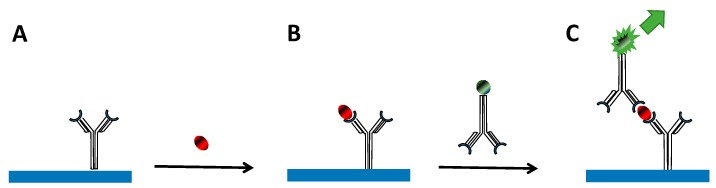
Schematic representation of immunoassay detection platform. (**A**) The capture substrate is modified with primary antibodies specific to the biomarker of interest; (**B**) the modified capture substrate binds and concentrates biomarkers from a sample; (**C**) secondary antibodies bind to a captured biomarker. Secondary antibodies are labeled to produce a measurable readout signal.

**Figure 2:**
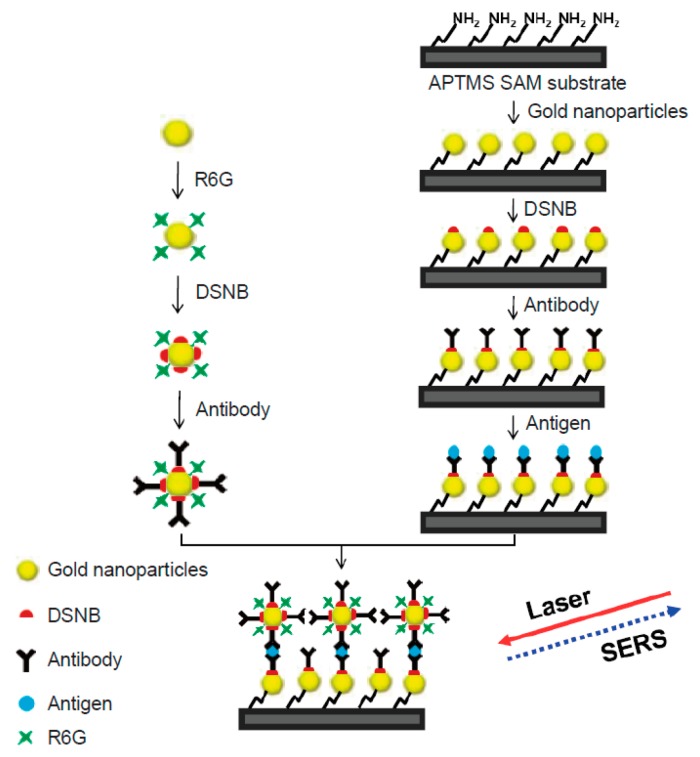
Sandwich-type immunoassay with SERS substrate prepared from AuNPs immobilized on the glass slide via 3-aminopropyltrimethoxysilane (APTMS) (scheme reprinted from [28]). Primary antibodies were attached to substrate bound golden nanoparticles via 3,3′-dithiobis[6-nitrobenzoic acid]bis(succinimide)ester (DSNB). Exposure of this substrate to the sample results in binding of prostate-specific antigen (PSA). A sandwich immunoassay forms between the immobilized primary antibody, PSA, and a secondary antibody conjugated to the reporter golden nanoparticles. The reporter nanoparticles are coated with Rhodamine 6G (R6G) dye in addition to antibodies. Illumination with laser light provides a strong SERS spectrum of R6G, proving the presence of prostate-specific antigen (PSA).

Immunoassay detection modality combines the ability of an antibody to recognize and bind a specific biomarker macromolecule from serum—a complex mixture of macromolecules. Multiple washing steps help to eliminate nonspecific detection as much as possible. Such specific immunoassay methods, which are referred to as “direct” detection, may feature either competitive or non-competitive format [1]. Competitive assay is more suitable for small biomarker molecules. A typical non-competitive assay is performed in a “sandwich” format that needs a larger biomolecule capable of binding at least two antibodies. Figure 1 schematically represents a typical sandwich immunoassay. Such an immunoassay generally involves two steps: (1) A capture surface modified with specific antibodies that bind and concentrate a biomarker from a sample (Figure 1A,B); (2) A secondary antibody is introduced that binds specifically to a captured biomarker molecule (Figure 1C). This secondary antibody is modified to provide a measurable readout – signal. Signal intensity reports on the biomarker concentration since the labeled antibody will not bind in the absence of the biomarker. There are two key elements in any sandwich immunoassay: (1) specificity afforded by marker specific antibody pair and (2) a readout strategy that provides high sensitivity with low noise to the assay. The main focus of this article is to explore the advantages of the SERS readout strategy for creating sensitive and robust detection platforms. 

## 3. Surface-Enhanced Raman Scattering as Readout Strategy—Mechanism and Advantage

A critical feature of all immunoassays is the means to produce a measurable signal in response to the biomarker binding. As Figure 1 shows, sandwich immunoassays involve the use of the second antibody carrying some kind of a label. This label allows for detection of the amount of secondary antibody, which in turn reports on the amount of captured biomarker molecules. The label thus provides a readout strategy allowing for means of detection. Various readout strategies have been developed and implemented over the course of several decades. Examples of applied readout strategies are scintillation counting [2], fluorescence [3], chemiluminescence [4], electrochemical [5,6], enzymatic methods [7], and quantum dots [8,9]. The most common ones are Radio-Labeled Immunoassay (RIA) and Enzyme-Linked Immuno-Sorbent Assay (ELISA) due to their low cost and convenience of use. 

Traditionally, fluorescence-based detection systems have been widely used as diagnostic tools for immunoassays; however, these systems have several drawbacks, including a poor limit-of-detection (LOD) and photobleaching. Additionally, fluorescence limits multiplex detection capabilities where several biomarkers are detected at once. To overcome these difficulties, new readout strategies are being actively sought. One such novel readout strategy is Surface-Enhanced Raman Scattering (SERS), which seems to be very effective at overcoming the above limitations. 

At the heart of the SERS readout strategy is the Raman effect, which is an inelastic scattering of incident photons by a molecule upon illumination with electromagnetic radiation. Most of the incident photons are scattered by a molecule with the same frequency elastically via so-called Rayleigh scattering. A very small fraction of the incident photons, however, is scattered with the frequency different from that of the excitation source—inelastic scattering. The difference in frequency is indicative of energy spent on exciting vibrations of the molecule. This type of scattering is called Raman scattering. The efficiency of traditional Raman scattering is very small—only one in about 10^10^ of all the incident photons is scattered inelastically, and only these contribute to the intensity of the Raman scattering observed [10,11]. Such low efficiency of Raman scattering results in low signal intensities that are not enough for the immunoassay readout even with the use of extremely intense excitation sources [12,13]. Fortunately, the efficiency of the Raman scattering can be enhanced when the molecules are in close proximity to surfaces of coinage metals such as Au, Ag, and Cu. In this case, the number of Raman-scattered photons is significantly amplified and reported to reach factors up to 10^14^ for certain systems [14]. 

Such a strong amplification of the Raman signal allows for single-molecule detection, as has been reported by several research teams [15,16]. Only certain configurations result in such high-intensity Raman signal [17]. For example, a gold–gold junction in a “mirror-like” configuration has been identified as capable of providing sufficient amplification to detect a Raman signal from a single molecule [18]. An additional enhancement is a result of coupling between continuous metal films and plasmonic particles [19]. Conveniently, such configuration is also suitable for sandwich-type immunoassays for detection of biomolecules. The design and advantages of such configuration for sensing applications are discussed in the next section. 

There are two mechanisms generally described in relation to the SERS effect—electromagnetic and chemical. It is widely accepted that scattering enhancement is mainly due to the dramatic increase in the electromagnetic field at the metal surface because of the surface plasmon resonance excitation [20]. Such huge enhancement factors allow for an effective and low-level readout strategy [21,22,23]. 

Excitation of the plasmon resonances is typically localized and occurs preferentially in the sharp nanoscale features of plasmonic materials such as coinage metals, as well as in the gaps between metal surfaces. The enhancement factor contributing to SERS depends on the magnitude of the localized electromagnetic field as ~E^4^ [24]. The chemical mechanism also contributes to signal enhancement for certain molecules, with charge transfer in molecules at the metal–molecule interface [25,26]. Regardless of the mechanism, SERS strategy provides a “fingerprint-like” Raman spectrum that is unique to the excited molecule used. The molecules utilized in SERS-based detection strategies are termed Raman labels or Raman tags. These features have allowed the Raman signal in the SERS format to rival and even exceed fluorescence’s performance as the readout strategy. Major advantages of Raman readout strategy over fluorescence assay are: (A) reduced susceptibility to photobleaching, a major problem of fluorophore-based assays and (B) Raman spectral bandwidths are significantly narrower than fluorescent band emissions. The narrow nature of Raman bands is an important feature providing great potential for developing multiplex detection capabilities.

## 4. SERS-Based Immunoassay Sensors

Similar to the conventional sandwich immunoassays illustrated in Figure 1, a SERS-based immunoassay uses three steps in sensor preparation. While the general principles of this strategy are the same, there is one critical modification—the readout signal is surface-enhanced Raman scattering. For example, a sandwich immunoassay format that uses SERS as a readout strategy was introduced for the quantitative detection of prostate-specific antigens (PSA) biomarkers by using Raman-labeled nanoparticles [27]. In this assay, a neutral substrate (a polystyrene microtiter plate) was coated with primary antibody. Exposure of this substrate to a solution containing different concentrations of biomarkers resulted in binding of the biomarkers to the modified substrate. After nanoparticles labeled with secondary antibodies were introduced, the amount of captured biomarkers is reflected in the intensity of the SERS signals.

In another configuration of sandwich-type SERS immunoassay, the substrate was prepared using a layer of AuNPs immobilized on the glass slide modified with 3-aminopropyltrimethoxysilane (APTMS) [28]. Primary antibodies were attached to golden nanoparticles, which were, in turn, immobilized on a substrate. Similarly, this modified substrate was exposed to a solution containing prostate-specific antigen (PSA). A sandwich immunoassay was formed between the immobilized primary antibody, PSA, and the secondary antibody conjugated to golden nanoparticles coated with Rhodamine 6G dye (Figure 2). A strong SERS spectrum was observed only in the presence of PSA, providing the femtomolar sensitivity of the assay.

**Figure 3:**
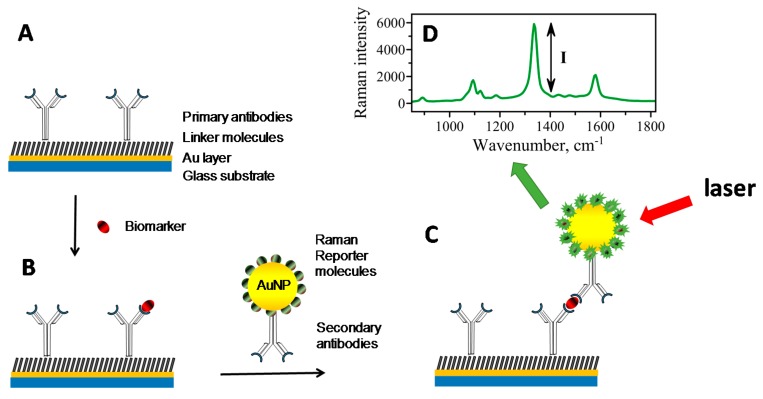
SERS-based sandwich immunoassay. (**A**) Capture substrate constructed using a thin smooth layer of gold on a glass slide. Thiolated linker molecules allow for covalent attachment of primary antibodies to golden layer. (**B**) Primary antibodies capture biomarker molecules from a sample. An introduction of secondary antibodies carrying AuNP–SERS nanotags modified with Raman reporter molecules and secondary antibodies completes the formation of sandwich immunoassay. (**C**) Laser light exciting plasmons in AuNP stimulates enhanced Raman scattering in Raman reporter molecules attached to AuNP. Plasmonic coupling between the AuNP and Au layer further contributes to signal enhancement. (**D**) The intensity of the SERS signal is detected and analyzed based on the amount of biomarker bound. Adopted and modified with permission from reference [40].

A SERS readout strategy has been demonstrated to have considerably high immunoassay response in the presence of antigen [12]. The detection limit for such a technique was reported to be 1 pg/mL of PSA in human serum [13]. The platform, as shown in Figure 3, utilizes a sandwich immunoassay format for the detection of biomarkers. There are three critical components: (1) a capture surface (Figure 3A); (2) AuNP—SERS nanotags (Figure 3C); and (3) a device capable of measuring Raman readout signal (Figure 3D). Both capture surface and SERS nanotags are modified with antibodies capable of specific binding of a molecule of interest—an analyte/biomarker. In the process of sample testing, the capture surface first binds and concentrates analytes from the sample (Figure 3B). Then, the exposure of this substrate to a solution containing SERS nanotags results in their binding to the capture surface via antibody–antigen interaction in a sandwich format (Figure 3C). Subsequent analysis of the sample with the laser light produces a readout signal, a SERS spectrum—which is a “fingerprint” profile that is unique to the Raman reporter molecules (Figure 3D). A sandwiched format has been a major focus of the developments in SERS-based immunoassays. Various kinds of biomarkers have been used to demonstrate the efficacy and sensitivity of the SERS-based assays. These biomarkers (Table 1) included immunoglobulin (IgG) antigens [12,29,30,31], feline calicivirus (FCV) [32], prostate-specific antigen (PSA) [13], protein A [33], hepatitis B virus [34], *Mycobacterium avium* subsp. Paratuberculosis [35], Alpha 1 fetoprotein (AFP) [36], and porcine circovirus type 2 [37]. It has also recently been demonstrated that SERS-based immunoassay outperforms conventional platforms such as enzyme-linked immunosorbent assay (ELISA) and radioimmunoassay (RIA) in the detection of MUC4 cancer biomarker [38,39]. Also, a recently developed SERS-based nano-immunoassay with improved performance surpasses the analytical capabilities of both ELISA and RIA [40]. The nano-immunoassay (unlike conventional ELISA and RIA) not only detects low levels of mucin biomarkers but is also capable of differentiating samples of cancer patients from those of healthy individuals [40]. The above studies demonstrated that SERS-based platforms are highly sensitive and reproducible immunoassays. 

## 5. Capture Substrate

The two most critical components of the SERS-based immunoassay are the capture substrate and the Raman labels/tags that produce the SERS signal. A capture substrate is a solid surface that allows for capturing the biomarkers. One might think of this substrate as an inert carrier of the capture antibodies with the sole goal of extracting biomarkers from a sample. In fact, this is the case for the majority of the sandwich immunoassays, including ELISA and RIA. In the classical protein assays, solid substrates at the bottom of the sandwich structure serve as the protein immobilization platforms. A lot of effort has been put towards choosing and optimizing various types of different surfaces in recent years. It has been reported that the SERS effect can be attenuated by various substrates [44]. Thus far, assays have been made using many different solid substrates including glass slides, microtiter plate wells, and filter supports. Of particular importance are the SERS-active substrates that can further increase the detectable Raman signal. Such SERS-active solid substrates have recently been utilized in constructing immunoassays that included (1) roughened metal substrates; (2) substrates assembled with silver nanoparticles; and (3) substrates assembled with gold nanoparticles. The SERS-active substrate is a functional participant in the detection process as it provides higher SERS enhancement capacity.

Higher enhancement capacity further increases the sensitivity of the SERS-based immunoassay. In a SERS-based immunoassay, the significance of the capture substrate is substantiated by the additional plasmonic coupling that SERS golden nanotags achieve after the binding event due to a close proximity of the metal–metal interface. The enhanced electromagnetic field is not only excited around the gold nanoparticles but is also generated and localized on the surface of the SERS-active substrate [45,46]. Comparison of the gold surface with a simple inert glass substrate indicated that a gold capture substrate is critical for achieving high-intensity Raman signals. The intensity increase is due to electromagnetic coupling between the plasmons of the particle and the underlying gold substrate [47].

Attaining controllable enhancement is also a key to using SERS-based immunoassay for quantitative characterization of biomarker concentration. In the immunoassay format, golden nanotags interact with the underlying surface due to its close proximity after immunoassay assembly. Theoretical and experimental studies showed that the efficiency of plasmon coupling between gold nanoparticles and the metal surface is dependent on the distance between contacting surfaces [47,48,49]. Larger electric field magnitudes in a golden particle–substrate junction, so-called “hot spots”, provide a higher SERS signal. Consequently, the SERS signal is very sensitive to the efficiency of the plasmonic coupling between nanotags and the underlying metal surface. For example, the distance between nanotags and a golden surface has been demonstrated to critically influence SERS signal due to the shift of the Surface Plasmon Resonance (SPR) maximum. As much as a 46 nm shift has been observed when the separation distance between a 60-nm nanoparticle, and the golden surface was reduced from 2.3 nm to 1.1 nm [46]. The extent of coupling between a nanoparticle and the surface is generally dependent on the ratio of the particle diameter to the separation between the particle and substrate (D/A) [48]. Thus, not only nanoparticle size but also gap distance can serve as a parameter for manipulation of SPR [46]. 

It also appears that surface morphology and material play an important role in generating surface enhancement. A golden star-shaped surface produces a much larger SERS as compared with a relatively flat surface [50]. In principle, any irregularities of the surface may create hot junctions, intensifying the signal. A much higher signal provides higher sensitivity for the detection in the immunoassay. A trade-off in such a design is uncontrollable signal intensification, which may make calibration challenging. A better approach for the SERS-based immunoassay detection platform is to synthesize the components with controllable shapes to ensure the reproducibility of the assay. One of the components in the immunoassay is gold nanoparticles, where homogeneity in size is important for the signal amplification [46]. Currently available commercial nanoparticles have a very high degree of homogeneity in terms of particle diameter with no need for further improvement. Yet another critical component of the assay is gold surface topography. The nanoscale characteristics, smoothness in particular, are important contributors to the SERS enhancement. 

A wide variety of SERS substrates has been actively investigated in recent years. These substrates include simple roughened noble metal surfaces [9] and noble metal colloids. Recently, more complex and optimized plasmonic systems have been explored for the purpose of analyte detection. These systems have been prepared by the assembly of nanospheres that provide remarkable SERS enhancement [51,52,53], template directed deposition [54,55], photolithography [56], a focused ion beam (FIB) [57], and electron-beam lithography [58,59]. These various modern methods allow for better control over the nanoscale features of the nanostructures. While major effort in preparation of SERS active substrates has been directed at creating substrates with higher enhancing capabilities, reproducibility is a more important issue in the SERS-based immunoassay.

A robust and reproducible way of creating a gold substrate has been previously introduced [46,48]. This method was termed the template-stripped gold (TSG) substrate. The biggest advantage of this substrate is the gold-based surface with a uniformly deposited smooth layer that is created freshly. In the preparation of a TSG substrate, the glass chips are gently detached from the silicon wafer. This results in a smooth gold surface on the glass chip [48]. It has recently been demonstrated that the SERS-based immunoassay dramatically benefits from using atomically smooth mica as the template, which further increases the reproducibility of the assay [40]. The atomic smoothness of a mica template gold substrate eliminates contributions from uncontrollable locations of sharp surface asperities or “hot spots” that contribute largely to SERS signal enhancement.

Another factor to consider in capture substrate is the ability to easily and effectively immobilize specific capture antibodies. Gold provides convenient thiol-based chemistry. First, we must find a coupling agent that can bind to gold as well as react with primary amines of antibodies to covalently attach them to the capture surface. For example, an easy and convenient way to attach antibodies using DSP was found to be a reliable linking agent [48]. Another example involves a heterobifunctional linker, hydrazide-polyethylene glycol-dithiol, that attaches a nonbinding region of the antibody to the gold nanoparticle and maximizes antibody functionality after attachment [60].

Generally, the reproducibility factor overwhelms the sensitivity factor such that instead of chasing the ultimate limit of sensitivity the focus is more on making a platform capable of reliable and reproducible measurements. Reproducibility becomes very important for detecting low levels of biomarkers for diagnostic purposes.

## 6. SERS Nanotags

**Figure 4:**
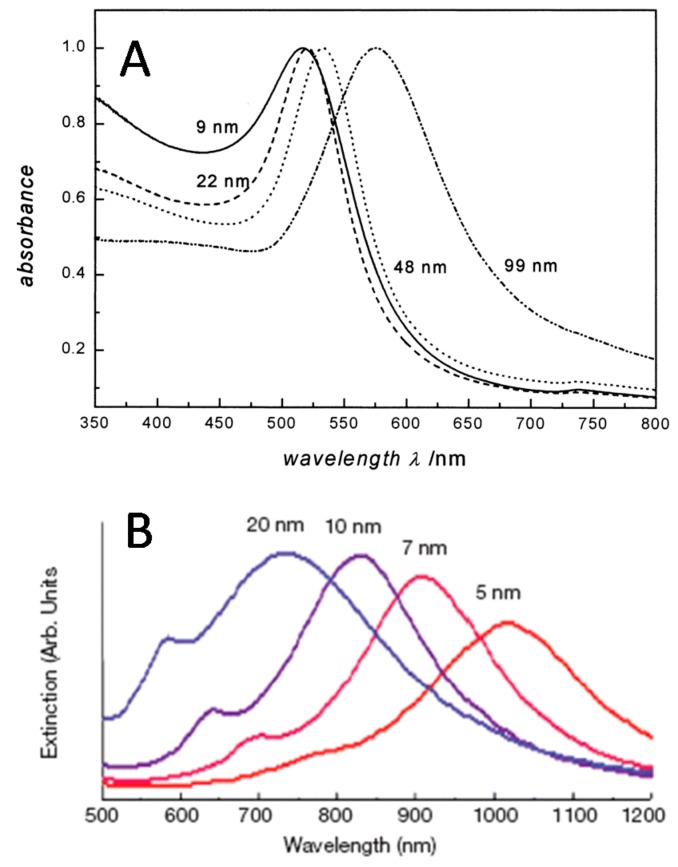
LSPR peak dependence on shape and composition of plasmonic tags. (**A**) Spherical AuNP (with permission from [66]); (**B**) Au–Ag nanoshells (with permission from [67]).

The SERS effect takes place near the surface of coinage metals [61]. In particular, silver and gold have wide use in SERS due to the convenience of surface plasmon excitation and high scattering efficiency. Collective oscillation of the free electrons in a metal particle induced by the oscillating electromagnetic field of the light reaches a maximum at a specific frequency called surface plasmon resonance (SPR) [62,63,64,65]. Surface plasmon resonance can be detected with a UV–Vis spectrometer as it induces a strong light absorption. The extinction maximum of spherical nanoparticles of the most commonly used plasmonic metals, silver, and gold, appears in the blue to green part of the electromagnetic spectrum. The position of the plasmon peak is dependent on a number of parameters, for example, metal type, size, and shape of nanostructures, as well as the dielectric function of both the metal sphere and the surrounding medium [63]. Figure 4A shows the dependence of the plasmon peak on the size of spherical gold nanoparticles. For nanostructures with a complex composition, for example gold/silver composite nanoshells, the plasmon peak shifts to longer wavelengths (Figure 4B). Since the maximum of the plasmon peak can be adjusted by tweaking nanostructure’s shape, material, and size, it is possible to shift the plasmon frequency to match the excitation laser wavelength. For example, when the shape of nanostructures is changed from spheres to rods, the SPR band has an additional peak in the NIR region corresponding to electron oscillations along the long axis, referred to as the longitudinal band. A larger red shift of the longitudinal band is observed for increasing aspect ratios (length to width) and respective color change of colloid from blue to red. Such a large degree of tunability provides versatility to the noble metal nanostructures in SERS nanotag preparation. 

The typical design of the SERS nanotags combines metallic (either silver or gold) nanostructures and specific organic Raman reporter molecules attached to or close to the surface of the particle. Most often gold nanostructures are used for SERS tags construction due to their relatively high stability and long shelf life. Several groups have used silver as their metal of choice for nanotag preparation. The nanotags utilize the effect of surface plasmon excitation to enhance the Raman signal of Raman reporter molecules (RRM). The large number of RRMs and the surface enhancement effect produce a strong, characteristic Raman signal that can be used for detection of small amounts of biomarkers. Typically, nanotags also require a protective layer—which will be discussed in the next section.

There are several different approaches that have been introduced to generate SERS nanotags. Primarily, the shape of nanotags receives special attention. Examples include: spherical nanoparticles [12,46], nanorods [68,69], nanoshells [70,71], nanostars [72], and multi-branched gold nanoparticles [37]. Excellent control over both morphology and composition of nanostructures has been achieved over the past two decades [73]. SERS capability has been experimentally demonstrated on various nanostructures including nanospheres, nanoshells, and nanorods [74]. There exists a large variety of shapes of nanostructures that can potentially be used in nanotag design [73]. Schutz et al. [72] synthesized multi-branched star-shaped golden nanostructures, which they used as SERS nanotags. Multiple sharp spikes act as lighting rods for the enhanced electromagnetic field. Also, the SPR band was shifted to a longer wavelength, thus avoiding the majority of autofluorescence signals. Functionalized nanostars have been successfully utilized as SERS nanotags for the detection of tumor suppressor p63 in the basal epithelium of benign prostate tissue. A similar strategy but different synthetic routes were used by Yuan et al. [75] and Luo et al. [76] to produce multibranched golden nanostructures as active SERS nanotags. A thorough study of morphology variations and its relation to shifts of the long plasmon band in the NIR region, thus tuning capabilities, has recently been presented [77].

Stars and multi-spiked tags, in contrast to spherical nanoparticles, provide larger enhancement of Raman signal, potentially contributing to higher sensitivity. Although a great deal of effort has been invested in developing multi-spiked nanotags, such geometry of tags contributes to the high variability of Raman signal enhancement. It is difficult to create a controllable number of hot spots that equally enhance the Raman signal of the Raman reporter molecules. One example of symmetric golden nanostars demonstrating a solution-phase method that yields a high degree of symmetry and monodispersity has been reported [78]. Such symmetric structures promoted superior SERS intensity and reproducibility as compared to asymmetric nanostars [78]. 

Other types of more complex nanostructures include nanoparticle dimers and multimers [79], nanoshells [80,81], core-shell [82], hollow gold nanospheres (HGNs) [83], and nanoparticle aggregates [84,85]. A modification of core nanoparticles with nanoparticles of smaller size [86], for example, AuNP-AuNP, AuNP-AgNP, has been also utilized [87]. Also, a modification of the core Au nanoparticle with other metals such as Pt or Pd has been shown to shift the plasmon peak to a longer wavelength, indicating that such modifications can be used to tune plasmonic and optical properties of SERS nanotags [88].

Although other geometries offer higher signal enhancement, the most reproducible form of SERS active nanoroughness is the spherical shape of metal nanoparticles. They can also be synthesized using easy and fast synthetic routes. Therefore, most research groups have focused on preparing nanotags with high SERS ability and homogeneous characteristics using spherical nanoparticles. 

By design, SERS nanotags combine metal nanoparticles with Raman reporter molecules and specific antibodies for biomarker recognition. Complex modification procedures involving the formation of self-assembled monolayers (SAM) have been introduced and optimized to increase both the readout signal and antibody surface coverage [48]. SAM modification allows for the following advantages in the nanotag design: (1) the highest number of Raman scattering molecules; (2) a uniform orientation of RRMs in the dense monolayers; and (3) a small gap between RRM and the metal surface. All these advantages contribute to a larger SERS signal upon a single binding event in the immunoassay. The optimized modification procedures also contributed to long-term stability, allowing for storage of prepared nanotags [48]. Additional protection strategies can be introduced to increase the nanotag stability. These protection strategies also aim at reducing the inherent limitation of SERS nanotag design, Raman signal deterioration upon prolonged laser illumination. One of the biggest advantages of SERS nanotags is that they allow for multiplex analysis of several biomarkers by introducing a unique RRM and an antibody specific to the targeted biomarker.

Next, we discuss Raman reporter molecules, protection strategies, and multiplexing, addressing advantages, limitations, and possible solutions of nanotag design that benefit SERS-based immunoassays. 

## 7. Raman Reporter Molecules (RRM)

Typically, SERS nanotags are constructed by immobilizing strong Raman active molecules with a large Raman cross-section on the surface of either silver or gold nanoparticles. Therefore, the sensitivity of the assay utilizing the nanotags will depend on the signal intensity generated by the RRMs. 

So far various different Raman active molecules have been utilized as RRMs. Novel Raman reporter molecules are being synthesized often to improve the functional abilities of RRM: their ability to form self-assembled monolayers, improved Raman signal intensity, and biocompatibility for in vivo imaging applications. 

The ability to form SAM layers contributes largely to the intensity and reproducibility of the SERS signal. Self-assembled monolayers are densely packed molecular layers that maximize the number of molecules in close proximity of metal surface and thus SERS signal generated by localized surface plasmon. SAM also improves molecular orientation variability and relative distance of a vibrating group to the metal surface. One of the most dependable molecules shown to form self-assembled monolayers is thiophenols [12]. In addition to thiol-gold chemistry, which is a simple and versatile route to surface modification [48], thiophenols are small and symmetric molecules that are easily identifiable with only a few characteristic Raman bands. Due to a small number of observable Raman bands, thiophenols are well suited for multiplexing, whereby several biomarkers are detected using the unique spectral signature of RRM. 

When dyes such as Rhodamine 6G, Cy3, Cy5, or Malachite Green are used as Raman reporter molecules, they experience an extra enhancement via Surface-Enhanced Raman Resonance Scattering or SERRS [89]. This effect contributes to higher sensitivity due to a larger observed signal intensity. However, dye molecules usually have many bands in their spectra, which complicates analysis and multiplexing. Fortunately, overlapping bands from different dyes can be differentiated by multivariate spectral analysis.

## 8. Protection Strategies

Nanoparticle-based SERS nanotags require protection and stabilization. The nanotags are usually modified with a large number of Raman Reporter molecules whose Raman signal is enhanced. Metal colloids and metallic nanostructures are usually stabilized with capping agents after they are synthesized to make them soluble in aqueous solutions. When these colloids are further modified with organic Raman Reporter molecules to produce SERS, the nanotag stability of such colloids may be compromised. SERS nanotags may become insoluble and aggregate as a result. Preventing a colloid solution from aggregation and improving nanoparticle storage requires the application of capping/stabilizing agents.

Various ligands have been employed as capping agents, for example citrate ions [90], tannic acid [91], surfactants with different lengths of the hydrocarbon chains [92], organic polymers such as polyvinylpyrrolidone (PVP) [93], polyethylene glycol (PEG) [94], or polymethyl methacrylate (PMMA) [95], branched polyethylenimine [96], biopolymers such as proteins [97], or polysaccharides [98]. All these agents need surface-seeking groups that make them stick to the nanoparticle surface. Introducing a multidentate polymer with multiple surface-seeking groups has been reported to be more beneficial in the colloid stabilization of gold or silver nanoparticles [99,100].

Advantages of polymer molecules also include a possibility for their subsequent modification at a free end, for example, conjugating nanoparticles with antibodies. Polymeric moieties could be introduced directly into RRMs to serve as stabilization agents [101]. Covalent attachment of PEG moieties—a short monoethylene glycol (MEG-OH) and a longer triethylene glycol (TEG-COOH)—to a Raman reporter molecule has been reported as an effective method of colloid stabilization and modification utilizing a terminal COOH group [101]. Such a strategy, where RRM itself is modified with the stabilizing agent, has been introduced to maximize the coverage of a metal nanoparticle surface with molecules producing a Raman signal. In general, a dense SAM monolayer benefits the observed intensity of the Raman signal due to a high amount of RRMs experiencing a surface enhancement effect. It has been suggested that aryl thiols are a good choice of RRMs due to their ability to form self-assembled monolayers [12,46] as well as the relative simplicity of their chemical structure, thus minimizing the number of Raman bands observed in a spectrum [74]. 

Schlücker et al. have introduced silica encapsulation as a protection strategy with adsorbed RRM as SERS tags [102,103]. Such a strategy not only has protective properties but also adds to the mechanical stability of metal nanoparticles and allows for long-term storage. 

In addition to the colloidal stability of metal nanoparticles, practical applications of SERS nanotags require the stability of the SERS signal when nanotags are exposed to continuous illumination of relatively high-power laser light [40]. Further, Raman reporter molecules may undergo photoinduced and thermal decomposition when exposed to long periods of intense laser light. An additional challenge is desorption of the RRMs from the particle’s surface. This leads to common problems in the SERS methodology including signal deterioration over time and an accumulation of broad peaks from carbonaceous contamination [40,45,104]. 

These problems associated with SERS limit the quantitative analysis of the readout signal in sensor applications and prevent the development of a reliable and quantitative SERS-based detection platform. Therefore, the protection and stabilization of the SERS nanotags is a key factor in developing reliable detection assays.

**Figure 5:**
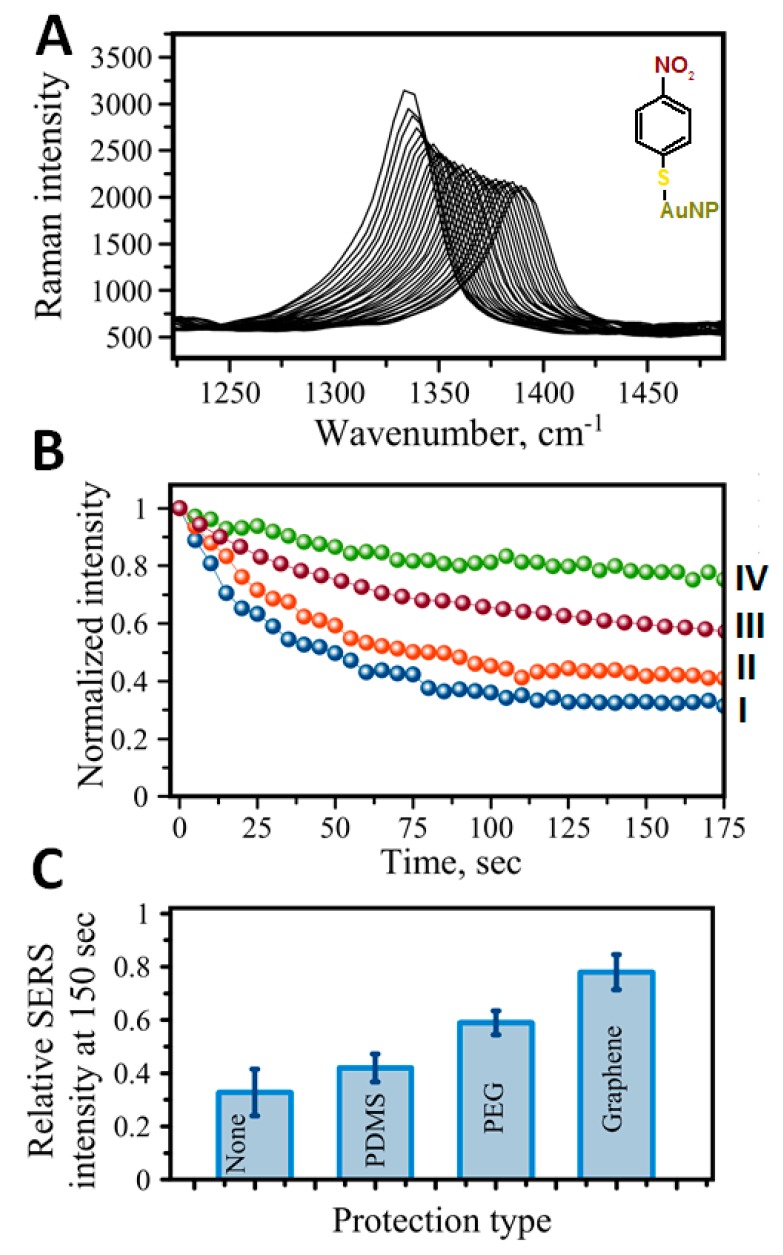
Protection of SERS-based immunoassay. (**A**) Time dependence of the signal intensity for the 4-nitrobenzenethiol used as a Raman reporter molecule. Raman peak at 1336 cm^−1^ corresponding to NO_2_ symmetric stretch vibration deteriorates with time under laser light exposure (spectra shifted for clarity). (**B**) A comparative graph showing the intensity of 1336 cm^−1^ band with time for different protective strategies: (I) no protection, (II) a layer of PDMS, (III) PEG-1000 Da coadsorbed with RRMs on Au nanotag, (IV) graphene monolayer applied on top of immunoassay addresses. (**C**) Comparison of intensities at 150 s time for protection shown in (**B**).

In order to increase the resistance of the RRMs to photodamage and improve the stability of the assay, a transparent protective coat of PDMS has been applied on top of the assay [40]. The intensity of the SERS signal declines over time due to RRM degradation upon laser irradiation (Figure 5A,BI). A PDMS overcoat improved the performance of the assay as the decline of the signal was observed to be much slower than that observed for the non-coated assay (Figure 5BII). Such coating also helped prevent the appearance of contaminating carbonaceous peaks. A single-layer graphene placed atop the assay increased the reliability of the assay even further as a protective layer in the SERS-based analytical assay. Only slight changes in SERS intensity have been observed within the first few minutes with graphene as a protection layer (Figure 5BIV). Graphene protection also resulted in high-quality Raman spectra with a much cleaner background and no amorphous carbon peaks, which is beneficial for quantitative analytical calculation.

Generally, protection aims at making nanotags more robust, especially preventing desorption of RRMs from nanoparticles and degradation of RRMs under intense laser light. We have recently tested the effect of SH-PEGm molecules on overall SERS signal stability upon intense laser irradiation. The presence of PEG is sought to form an encapsulating layer over Raman reporter molecules on the surface of the gold nanoparticle. Such an encapsulating strategy improves signal stability upon long exposure to intense laser light. Figure 5BIII shows the effect of PEG on the Raman signal stability of the nitrobenzene thiol reporter molecule. Figure 5C shows a comparison of the Raman signal (the nitro stretch at 1336 cm^−1^) after 150 s of laser irradiation for several different protection strategies. PEG of MW = 1000 Da co-adsorbed with Raman reporter molecules on SERS nanotags seems to be a better protective agent as compared to PDMS, approaching the efficiency of graphene protection. It has been hypothesized that local heating at the surface of gold nanoparticles [105] contributes the most to the photo/thermal decomposition of RRMs and their desorption from a locally heated surface of a nanoparticle [40]. Graphene is sought as the best protector due to its high transparency, gas impermeability, and high thermal conductivity—properties beneficial to its application in SERS-based immunoassays [40]. One advantage of PEG protection over graphene is the biocompatibility of the protected SERS nanotags, which makes them available for in vitro and in vivo applications [106]. 

Another interesting approach is to modify Raman reporter molecules to make them much more stable and resistant to intense laser irradiation. The first attempts with such an approach used modifications of triphenylmethine (TM) [107] and TM plus lipoic acid (LA) [108]. Modified reporter molecules not only have higher SERS intensity but also exhibit stability over time. TM-LA nanotags are also biocompatible and showed that it is possible to recognize cancer cells when further modified with antibodies specific for HER2 and EGFR cancer proteins [108].

## 9. Multiplexing with SERS Immunoassay

The Surface-Enhanced Raman Scattering (SERS) readout strategy offers the remarkable capability of multiplex detection of selected biomarkers. Detection of multiple biomarkers simultaneously provides a better strategy that improves the accuracy of disease diagnostics. Progression of disease is very often associated with the expression of multiple biomarkers that make up a “biomarker panel”. For example, recent reports indicate that no individual biomarker is ideal for the diagnosis and prognosis of pancreatic cancer [109]. Another example is a pattern of miRNA molecules associated with a specific type of cancer [110,111].

The discovery and validation of biomarker panels that would report on disease inception and progression are needed. Next, the development of detection methods that can detect multiple biomarkers in the panel with high clinical accuracy and specificity is required. The multiplex detection will also greatly contribute to a personalized diagnosis and prognosis since those can be features specific to an individual. Therefore, simultaneous profiling of multiple biomarkers (multiplexing) is beneficial for clinical applications of any promising detection platform. The following features of the SERS readout strategy make it very attractive in terms of multiplexing capabilities:

(1) The availability of reliable detection platforms with high sensitivity.
(2) The narrow width of the characteristic Raman bands observed in a spectrum for individual RRM.
(3) Single laser wavelength allows for concurrent excitation of many different Raman active reporter molecules.

**Figure 6:**
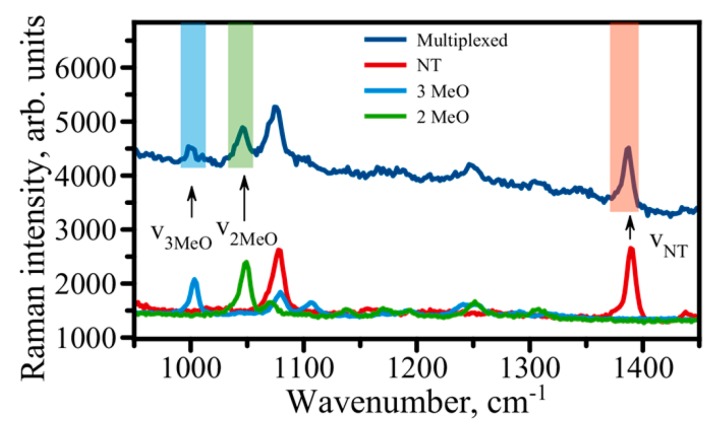
Proof-of-principle multiplex assay with three distinct Raman Reporter Molecules. Individual Raman spectra overlaid are shown in the bottom. The spectra correspond to RRMs: 3 methoxybenzenethiol (3MeO—light blue), 2 methoxybenzenethiol (2MeO—green), and naphtalenethiol (NT—red). The dark blue spectrum above is the spectrum taken on a multiplexed assay where 3MeO, 2MeO, and NT are used as three distinct SERS labels for multiplexing. Spectra are shifted for visual clarity.

SERS-based immunoassay possesses all these features, which makes this platform highly popular for multiplex biosensing, with several biomarkers being detected and quantified in a single run. It utilizes stable and robust SERS nanotags comprising a large number of Raman reporter molecules attached to the surface of metal nanoparticles. In addition to RRMs, each nanotag carries an antibody specific to a biomarker of interest. Thus, recognition of a biomarker and binding of nanotags in the immunoassay produces a characteristic spectrum of the RRM used that would report on the biomarker being detected. Spectral multiplexing can be achieved by using nanotags labeled with different RRMs with characteristic Raman spectra and antibodies corresponding to different biomarkers. Raman reporter molecules can be chosen such that there is very little spectral overlap due to the narrow bandwidth of vibrational Raman bands. For example, the relative simplicity and symmetry of the chemical structure of thiophenols produce a minimal number of narrow Raman bands in a spectrum, allowing for a higher number of labels to be detected simultaneously. Figure 6 shows a multiplex assay using three distinct variations of thiophenol-based RRMs. Obviously, there is only a small number of Raman bands in the 950–1500 cm^−1^ spectral window, which is a great advantage in using thiophenols. Also, only vibrations corresponding to aromatic C-C stretch at ~1090 cm^−1^ overlap, while there is no spectral overlap between the major peaks of these three RRMs. Thus, the three RRMs can be effectively used in multiplexing for the detection of multiple biomarkers. In such a multiplexed assay, AuNP are modified with one type of RRM and carry antibodies recognizing and binding one particular biomarker. Also, it is evident that there is plenty more spectral space for adding other thiophenol modifications for a greater order of multiplexing. In addition to a small number of Raman peaks for thiophenols, they are also capable of forming dense self-assembled monolayers (SAM) on a gold surface, which increases the number of molecules producing a Raman signal and also offers a similar orientation of molecules with respect to a surface, contributing to signal reproducibility. While only three thiophenols are shown in Figure 6, we have tested a library of 12 potential candidates thus far. The theoretical upper limit of SERS multiplexing has been estimated to be ~100 with the range of 10–30 various SERS labels to be quite realistic for microscopic applications [74]. 

**Figure 7:**
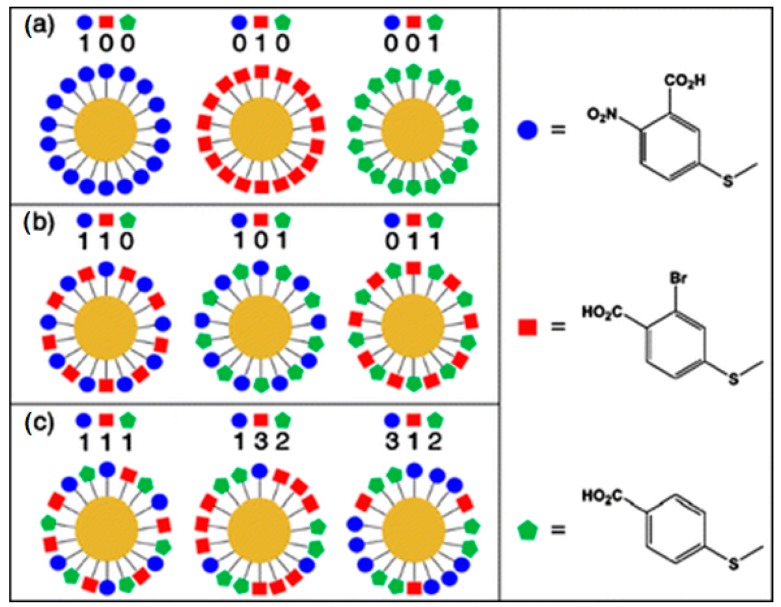
Left panel: schematic representation of different SERS tags, consisting of one-, two-, and three-component Self-Assembled Monolayers (SAMs) on spherical AuNPs. (**a**) One-component SAMs (with binary codes: 100, 010, 001); (**b**) two-component SAMs (with binary codes: 110, 101, 011); (**c**) three-component SAMs (with binary codes: 111, 132, 312). Right panel: structures of the molecules that were used for the SAM construction. Blue—5,5′-dithiobis(2-nitrobenzoic acid), red—2-bromo-4-mercaptobenzoic acid, and green—4-mercaptobenzoic acid. The identity of RRM as well as stoichiometry translated into intensity (1,2,3), can be used to construct distinct SERS nanotags. Reprinted from [112].

Furthermore, multiplexing capacity of SERS labels can be improved by applying the following strategy proposed by Schlücker and coworkers [112]. Briefly, they used mixed SAMs of three different Raman reporter molecules on the surface of the metal nanoparticle (Figure 7). The three RRMs used have similar Raman cross sections: 5,5′-dithiobis(2-nitrobenzoic acid) (DTNB), 2-bromo-4-mercaptobenzoic acid (BMBA), and 4-mercaptobenzoic acid (MBA). In a classic approach, with three different RRMs, only three distinct one-component SAMs are possible: 100, 010, and 001 (Figure 7a). The binary notation indicates the presence (1) or absence (0) of a particular RRM. If a two-component mixed SAM is created with three original RRMs, three additional combinations are possible: 110, 101, and 011 (Figure 7b). Seven total combinations can be produced with one, two, or three component SAMs: 100, 010, 001, 110, 101, 011, and 111. Furthermore, not only the type but also the stoichiometric ratio of RRMs in mixed SAM can serve as an additional parameter (Figure 7c). Therefore, using two parameters, type and stoichiometry, may provide a possibility for a very large number of spectroscopically distinct SERS nanotags [112].

Dye molecules have been also utilized as RRMs in the nanotags for multiplexing purposes. Although more peaks are generally observed in their Raman spectra, it is nevertheless possible to distinguish signals from several different dye molecules. Multivariate analysis eases such a nontrivial task when applied during post-acquisition analysis [113]. Several groups have successfully employed dye molecules as RRMs in multiplex assays for DNA detection. For example, Graham and co-workers have performed SERRS multiplexed experiments for discrimination of multiple DNA without the need for separation [114]. Quantitative detection of five labeled oligonucleotides without any separation was reported [115]. The sensitivity of the multiplex analysis is the same as that for the individual dyes and indicates that there is no compromise in the multiplexed format. 

Cao et al., have successfully employed dyes (Cy3, Cy3.5, Cy5, Rhodamine 6G, tetramethyl rhodamine, and Texas red) as Raman labels adsorbed onto small 13 nm AuNPs. Using these Raman labels, a highly sensitive and selective microarray platform was developed for the detection of DNA using oligonucleotide-functionalized nanoparticles as probes and silver staining for signal enhancement. Several different DNA target sequences were detected with ~20 femtomolar detection limit [116]. A number of recent reports have also demonstrated the capability of SERS multiplexing for in vivo as well as in vitro applications. 

## 10. Multiplex Detection in Vivo

SERS nanotags reactive against cancer-specific markers have also been tested for detection and localization of tumor tissues. Recent advances in SERS nanotags preparation allows for the tags to be biocompatible with tunable excitation to near-infrared where damage to biological tissues is minimal. Maiti et al. prepared SERS nanotags using lipoic acid-containing cyanine derivatives (Cy3LA and Cy5LA) to act as multiplex partners with a triphenylmethine Raman reporter (B2LA) under a single excitation wavelength [117]. Using these tags the authors demonstrated multiplex recognition of two different cancer cells, OSCC and SKBR-3, individual or co-cultured. In another study, in vivo multiplexing capability was demonstrated after injection of three BSA encapsulated nanotags with the following RRMs: CyNAMLA-381, Cy7LA, and Cy7.5LA. Equal amounts of these nanotags were injected through the tail vein of a living mouse bearing a tumor xenograft that expressed EGFR receptor. These nanotags conjugated with anti-EGFR antibody were specifically accumulated at the tumor site [69].

Multiplex SERS nanotags successfully detected cancer biomarkers EGFR (cell surface receptor for EGFR family members), CD44 (cell surface adhesion molecule and receptor for the glycosaminoglycan, hyaluronan), and TGFβRII (receptor for the anti-proliferative TGFb ligand) in an MDA-MB-231 breast cancer xenograft mouse model [118]. These biocompatible SERS nanotags were constructed by conjugation of SERS-active dyes MGITC, Cy5, and Rh6G to anti-EGFR, anti-CD44, and anti-TGFβRII antibodies adsorbed onto AuNPs followed by PEG modification to make them biocompatible. A mixture of SERS nanotags conjugated to specific antibodies (Rh6G-EGFR, MGITC-CD44, and Cy5-TGFβRII) were injected intratumorally to detect breast cancer cells under both in vitro and in vivo conditions. These SERS-nanotag-specific Raman spectra were observed both for tumor cells at 1120 cm^−1^ (Cy5), at 1175 cm^−1^ (MGITC) and at 1650 cm^−1^ (Rh6G,) as well as in orthotopically transplanted tumor tissues. Interestingly, antibody conjugated nanotags specifically targeting the three biomarkers exhibited maximum signal at 6 h and no detectable signal at 72 h. On the other hand, nanotags without antibodies showed no detectable signal after 6 h. Therefore, the study demonstrated that SERS nanotags are ultrasensitive nanoprobes for the multiplex detection of biomarkers under in vivo conditions [118].

Neng et al. used multiplex SERS for detection of the surface envelope and the capsid of two different viruses, West Nile virus (WNV) and Rift Valley fever virus (RVFV) [119]. Nanotags carried a specific antibody for each antigen and two different Raman reporter dyes, Nile blue (NB) and Infrared-792 (IR-792). Spectral signatures of the probes were detected after 785 nm laser excitation, suggesting the presence of target antigens. 

## 11. Concluding Remarks

Surface-Enhanced Raman Scattering has recently been utilized as a powerful readout strategy in the immunoassay-based biomarker detection platforms. Such platforms have been demonstrated to possess high sensitivity exceeding the performance of commonly used readout strategies. Significant driving forces for the use of SERS in the design of detection platforms are advantages that SERS possesses over other readout strategies. These advantages include higher detectable response to binding of a single biomarker as well as multiplexing capabilities due to the narrow nature of detected peaks from Raman Reporter molecules. Multiplexing with SERS provides the ability to detect and monitor a panel of biomarkers, making SERS-based detection platforms a more reliable approach in detection and monitoring disease progression. Furthermore, recent advances in nanomaterials and nanotechnology allow for better control of the quality and physical properties of the nanostructures, providing more reliable ways to construct SERS-based detection platforms. Recent developments, high sensitivity, and the ability for multiplexing of the SERS-based technology provide strong grounds to envision, in the near future, the construction of reliable point-of-care devices that will contribute to the fast-developing field of personalized medicine. 

## Figures and Tables

**Table 1:** List of biomarkers for which sandwich SERS immunoassay has been developed.

Biological Antigen	Limit of Detection (LOD)	Reference
Alzheimer’s Tau Protein	<25 fM	[41]
Prostate-specific antigen (PSA)	1 pg/mL (~30 fM)	[13,28]
Immunoglobulin (IgG) antigens	300 pg/mL	[12,29,30,31]
Metanephrine	<10 µM	[42]
Mucin 4 (MUC4)	33 ng/mL	[38,40]
*Mycobacterium avium* subsp. Paratuberculosis (MAP)	1000 MAP/mL	[35]
Hepatitis B virus	0.5 µg/mL	[34]
Feline calicivirus (FCV)	10^6^ FCV/mL	[32]
Carcinoembryonic antigen (CEA)	10 pg/mL	[43]
